# Position of chromosomes 18, 19, 21 and 22 in 3D-preserved interphase nuclei of human and gorilla and white hand gibbon

**DOI:** 10.1186/1755-8166-1-9

**Published:** 2008-04-29

**Authors:** Marina Manvelyan, Friederike Hunstig, Kristin Mrasek, Samarth Bhatt, Franck Pellestor, Anja Weise, Thomas Liehr

**Affiliations:** 1 Institute of Human Genetics and Anthropology, Kollegiengasse 10, D-07743 Jena, Germany; 2 Department of Genetic and Laboratory of Cytogenetics, State University, Yerewan, Armenia; 3 INSERM U847, Montpellier, France; 4 University of Montpellier I, Montpellier, France; 5 Department of Reproduction biology, CHU Montpellier, Montpellier, France

## Abstract

**Background:**

Even though comparative nuclear architecture studies in hominoids are sparse, nuclear chromosome architecture was shown to be conserved during hominoid evolution. Thus, it is suspected that yet unknown biological mechanisms must underlie this observation.

**Results:**

Here for the first time a combination of multicolor banding (MCB) and three-dimensional analysis of interphase cells was used to characterize the position and orientation of human chromosomes #18, #19, #21 and #22 and their homologues in primate B-lymphocytic cells. In general, our data is in concordance with previous studies. The position of the four studied human chromosomes and their homologues were conserved during primate evolution. However, comparison of interphase architecture in human B-lymphocytic cells and sperm revealed differences of localization of acrocentric chromosomes. The latter might be related to the fact that the nucleolus organizing region is not active in sperm.

**Conclusion:**

Studies in different tissue types may characterize more – potentially biologically relevant differences in nuclear architecture.

## Background

In the interphase nucleus, chromosomes are located in specific regions, which are called 'chromosome territories' [1-3]. Recently, own multicolor banding (MCB) based studies showed, that the chromosome shape is not lost in the interphase nucleus and one can even identify interphase chromosomes instead of only chromosome territory [4,5].

Both, chromosome size and gene density are discussed to have an important impact on the nuclear position of chromosomes. Small chromosomes preferentially locate close to the center of the nucleus, while large chromosomes can be found in the nuclear periphery of human fibroblasts [6,7]. On the other hand, Croft et al. (1999) [8] demonstrated a gene density-correlated radial arrangement of chromosomes in nucleus. Mainly gene-dense and early replicating chromatin, including the small human chromosome #19 with only 63 megabasepair (Mbp) in size but 27,9 genes/Mbp, can be found in the central part of the nucleus, while gene-poor and later replicating chromatin, like the human chromosome #18 (HSA #18) with a similar size like HSA #19 (77 Mbp and 8,7 genes/Mbp) is located in nuclear periphery [8]. This nuclear topological arrangement was exemplarily proven to be conserved during primate evolution over a period of about 30 million years: conservation of gene-density-correlated arrangement of human homologous chromosomes #18 and #19 has been shown in New World and Old World monkeys [9,10].

In this study the first comparative MCB-based analysis of interphase chromosomes was performed in B-lymphocytes of *Homo sapiens *(HSA), *Gorilla gorilla gorilla *(GGO) and *Hylobates lar *(HLA). Previously, we showed that human MCB probe sets can be applied successfully in GGO and HLA metaphase chromosomes [11,12]. While in GGO the homologues of the four selected human chromosomes are conserved as single chromosomes without additional rearrangements [11,13] in HLA rearrangements took place [8,12]. In GGO chromosomes GGO #16, GGO #20, GGO #22 and GGO #23 are completely homologous to HSA #18, HSA #19, HSA #21 and HSA #22, respectively. In HLA entire HSA #18 homologue is 'translocated' to the homologous segment of HSA 1p32-1q22 to form HLA #5. HSA #19 is distributed into 5 different parts on the chromosomes HLA #10, HLA #14 and HLA #16. HSA #21 and HSA #22 are parts of a HLA #15 and HLA #8, respectively. HLA #8 is homologous to parts of HSA #9, HSA #16 and HSA #22. HLA #15 contains parts homologous to HSA #15 and HSA #21 [12].

Here a combination of MCB technique [14] with suspension-fluorescence *in situ *hybridization (S-FISH) [15] allowed to perform three-dimensional (3D) studies for orientation and position of interphase chromosomes in B-lymphocytes of three hominid species.

## Results and Discussion

### MCB studies combined with S-FISH

**Figure 1:**
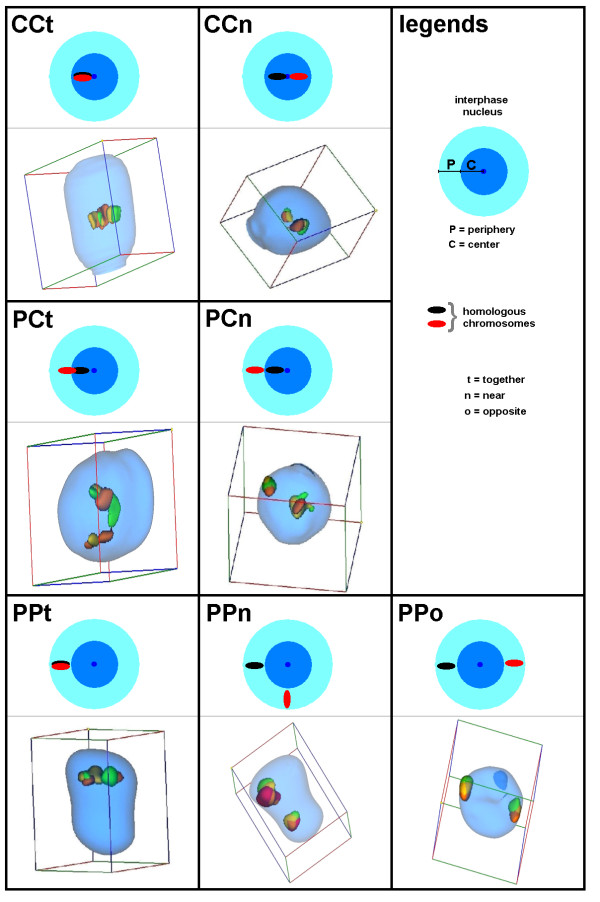
Schema of localization of chromosomes in different positions in the interphase nucleus. Abbreviations see legend in the figure and legend of Table 1.

**Figure 2:**
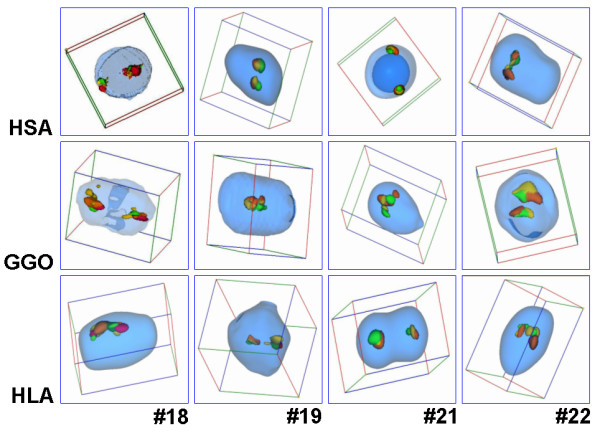
Typical results obtained after application of MCB probe sets for HSA #18, HSA #19, HSA #21 and HSA #22 in interphase nucleus of HSA, GGO and HLA.

**Figure 3:**
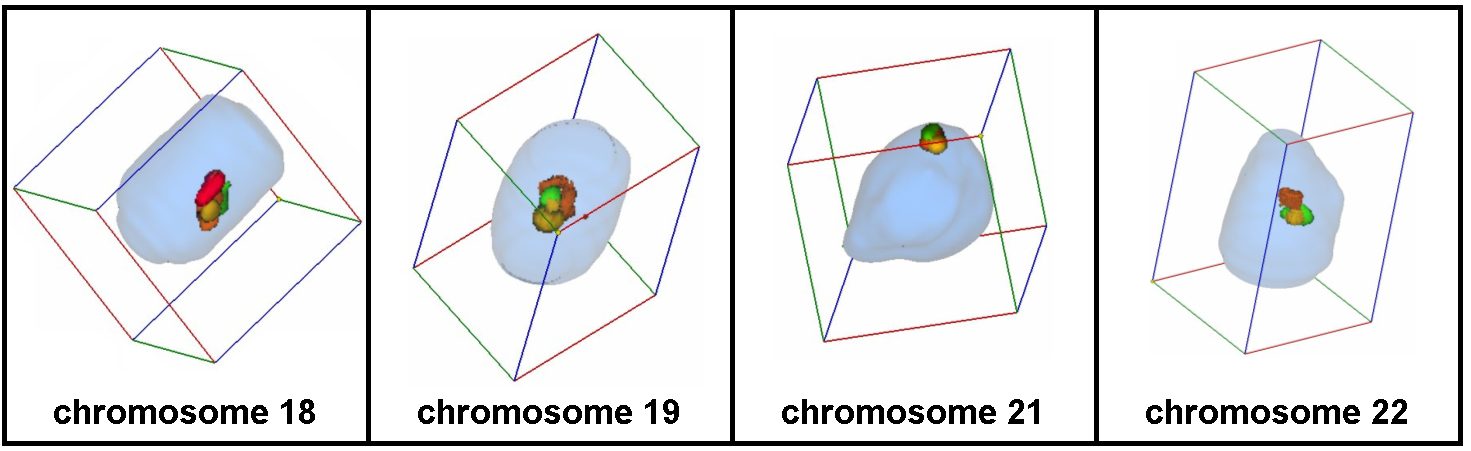
MCB probe sets for HSA #18, HSA #19, HSA #21 and HSA #22 applied in sperm derived from a normal human male.

Here we present the first MCB-based study on three-dimensionally preserved interphase nuclei derived from B-lymphocytes and sperm (Figs. 1, 2 and 3). Previously, comparable MCB-studies were performed on flattened nuclei with the known disadvantages of possible artifacts due to transformation of a spherical into a pancake-like object. Nonetheless, also important findings on decondensation of chromosomes during interphase could be obtained [4,5]. While it was initially not possible to apply MCB probe sets [12] in S-FISH [15], this problem was now successfully solved [16]. However, the comprehensive evaluation of one single interphase nucleus, including image acquisition, processing and analysis lasts about 4–5 hours using Cell-P software (Olympus). Thus, the number of investigated nuclei had to be restricted to 30 per tissue and/or species in this study.

### Position of individual chromosomes in B-lymphocytes of HSA, GGO and HLA

The evaluation of the molecular cytogenetic results was done as described in the Method-part (see below). Position and distance of homologous chromosomes in the interphase nucleus was determined as peripheral (P) and central (C). Thus, either both homologues were located in the center (CC), in the periphery (PP) or both, in periphery and center (PC). The localization of the homologue chromosomes to each other, when located in the periphery was estimated as 'close together' (t), 'near by each other' (n) or 'on the opposite sides of the nucleus' (o) – see Figure 1.

The obtained results for the position of HSA chromosomes #18, #19, #21 and #22 in B-lymphocytes of HSA and of their corresponding homologous regions in GGO and HLA are summarized in Tables 1 and 2. Below the results for each of the four studied chromosomes are reported and discussed. Overall, there were no significant differences in the localization of the studied chromosomes in the three different species for B-lymphocytes.

**Table 1:** Position of the chromosomes 18, 19, 21 and 22 in B-lymphocytes of HSA, GGO and HLA

species	HSA chr.	position of individual chromosomes			
		
		P		C	
		
		quantity	(M ± m)%	quantity	(M ± m)%
HSA	#18	51	**85.0 ± 4.6**	9	15.0 ± 4.6
GGO		52	**86.7 ± 4.4**	8	13.3 ± 4.4
HLA		53	**88.3 ± 4.2**	7	11.7 ± 4.1
HSA	#19	7	11.7 ± 4.2	53	**88.3 ± 4.2**
GGO		9	15.0 ± 4.6	51	**85.0 ± 4.6**
HLA		11	18.3 ± 5.0	49	**81.7 ± 5.0**
HSA	#21	49	**81.7 ± 5.0**	11	18.3 ± 5.0
GGO		42	**70.0 ± 5.9**	18	30.0 ± 5.9
HLA		49	**81.7 ± 5.0**	11	18.3 ± 5.0
HSA	#22	36	**60.0 ± 6.3**	24	**40.0 ± 6.3**
GGO		34	**56.7 ± 6.4**	26	**43.3 ± 6.4**
HLA		32	**53.3 ± 6.4**	28	**46.7 ± 6.4**

Position of the four studied chromosomes in 3D-preserved interphase nuclei derived from B-lymphocytes of *Homo sapiens *(HSA), *Gorilla gorilla gorilla *(GGO) and *Hylobates lar *(HLA). The position of 60 individual chromosomes was determined, each. Abbreviations: C = center; chr. = chromosome; M = Mean; m = Standard Error; P = periphery; see as well Figure 1.

**Table 2:** Orientation of the four studied chromosome pairs in HSA, GGO and HLA

species	HSA chr.	position of homologue chromosomes to each other									
		
		CC			PC			PP			
		
		CCt	CCn	**CC%**	PCt	PCn	**PC%**	PPt	PPn	PPo	**PP%**
HSA	#18	3	0	10.0	0	3	10.0	9	7	8	**80.0**
GGO		2	1	10.0	0	2	6.7	9	8	8	**83.3**
HLA		1	0	3.3	0	5	16.7	7	8	9	**80.0**
HSA	#19	18	7	**83.3**	0	3	10.0	1	0	1	6.7
GGO		17	6	**76.6**	3	2	16.7	0	1	1	6.7
HLA		18	6	**80.0**	0	1	3.3	1	1	3	16.7
HSA	#21	0	2	6.7	0	7	23.3	3	6	12	**70.0**
GGO		2	2	13.3	1	9	33.3	6	8	2	**53.4**
HLA		3	1	13.3	1	2	10.0	2	12	9	**76.7**
HSA	#22	7	2	**30.0**	1	5	20.0	6	7	2	**50.0**
GGO		2	9	**36.7**	0	4	13.3	5	9	1	**50.0**
HLA		8	3	**36.7**	5	1	20.0	7	5	1	**43.3**

The position of 30 chromosome pairs was determined, each. Abbreviations: C = center; CC = both homologues located in center; chr. = chromosome; n = homologue chromosomes located 'near by each other'; o = homologue chromosomes located 'on the opposite sides of the nucleus' (o); P = periphery; PC = both homologues located in periphery and center; PP = both homologues located in periphery; t = homologue chromosomes located 'close together'; see as well Figure 1.

### Chromosomes #18 and #19

According to our results (Tables 1 and 2) chromosome #18 is located in the periphery of the interphase nucleus in all three studied species: 85.0 ± 4.6% up to 88.3 ± 4.2% of studied #18 were located marginal. The localization of #19 was determined to be central in HSA, GGO and HLA with 81.7 ± 5.0% to 88.3 ± 4.2%. Thus, the position of #18 and #19 in nuclear architecture significantly differs from each other (for HSA t = 11.77; for GGO t = 11.26; for HLA t = 10.72; for all compared groups df = 118, p = 0.001). Hence, our data is in concordance with the results of [8] who suggested as a reason for that difference the divergence in gene density in these two chromosomes. The gene-density correlated radial arrangements of #18 and #19 were conserved during primate evolution, as previously shown [9].

For the analysis of position of homologous to each other only the data from the peripheral part of nucleus can be taken into account – thus, for chromosomes located in the center of the nucleus, like #19 (and #22) no such analysis was performed. For positions of homologous #18 to each other (Table 2) there was no significant differences in localization "PPt", "PPn" and "PPo". Thus, arrangement of human 18 homologue chromosomes to each other has random way in all three studied species (ANOVA-test for – HSA: F = 0.171, p = 0.843; – GGO: F = 0.054, p = 0.946; – HLA: F = 0.171, p = 0.843).

### Chromosome #21

According to [6,7] chromosome #21 preferentially locates close to the center of the nucleus. The authors there postulate that this is due to the fact that acrocentric chromosomes carry nucleolar organizer regions on their short arms and the nucleolus is generally located in the inner nuclear space. Bolzer et al. (2005) [6] demonstrated that the distance between chromosomal territories of homologues acrocentric chromosomes is significantly smaller than the mean distance for five largest chromosomes. In contrast, they showed as well that the distance between chromosomal territories of homologues acrocentric chromosomes was not significantly different from the mean distances for the other small chromosomes.

In the present study the arrangement of #21 in interphase nuclei is non-random. In contrast with previous studies [6,7], human homologues chromosomes #21 mostly localized in the periphery of the nuclei in 70.0 ± 5.9% up to 81.7 ± 5.0% (χ^2 ^= 55.6, df = 1, p = 0.001). Thus, the preferentially localization of #21 in the peripheral part of nucleus could be explained by gene-density correlated arrangement: at a size of 33.5 Mb chromosome #21 contains about 225 genes, which is two times less than in chromosome #22 of approximate the same size. Moreover, in HLA the homologous region to HSA #21 is not located on an acrocentric chromosome.

The analysis of homologous #21 localized in peripheral part of the nucleus to each other showed that PPt and PPn compared to PPo is not significantly different for HSA (t = 0.816, df = 58, p = 0.418) and HLA (t = 1.348, df = 58, p = 0.1839)but for GGO (t = 3.928, df = 58, p = 0.001). Thus, only GGO #21 behaves like postulated for an acrocentric chromosomes, as homologous ones mostly localized 'together' and 'nearby'.

### Chromosome # 22

The observed position of #22 in nucleus of B-lymphocytes is different from position of #21. While the #21 mostly localized in the peripheral part of nucleus, #22 allocated more equally in the territory of nucleus. Differences in position of these chromosomes in nucleus are significant (χ^2 ^= 5.97, p < 0.015). In the present study #22 was localized to about 50% in the peripheral and to about 50% in the central part of the nucleus for all three species (Table 1). This data is in discordance to [6] demonstrating that acrocentric chromosomes preferably locate close to nuclear center. Regarding the orientation of homologous #22 to each other, in all three species they tend to be co-localized in 87% to 93% of the cases. Comparing PPt and PPn with PPo for significant differences were observed (HSA: t = 3.62, df = 58, p = 0,001;GGO: t = 4.477, df = 58, p = 0.001; HLA: t = 3.849, df = 58, p = 0.001. These results are similar with orientation of #21 in GGO (Table 2).

### Position of individual chromosomes in B-lymphocytes and sperm of HSA

As shown in Table 3 localization of chromosomes #18, #19 and #21 in human B-lymphocytes and sperm is similar – however, this is not the case for chromosome #22. In sperm #22 is located in the center of the nucleus, i.e. according to its gene-density. It can be speculated that this is due to the fact that in genetically inactive sperm no nucleolus is formed. Thus, it could be postulated, that the chromosomes can here be arrange only according to gene density. In genetically active cells this primary order would then be disrupted by the fact that acrocentric chromosomes are attached to the more peripherally located nucleolus by their short arm carrying the nucleolus organizing regions. However, further studies have to be preformed to prove this suggestion.

**Table 3:** comparison of B-lymphocytes and sperm

HSA tissue	HSA chr	P [%]	C [%]
B-lymphocytes	#18	**85**	15
sperm		**77**	23
B-lymphocytes	#19	12	**88**
sperm		20	**80**
B-lymphocytes	#21	**82**	18
sperm		**67**	33
B-lymphocytes	#22	**60**	**40**
sperm		17	**83**

Position of the four studied chromosomes in 3D-preserved interphase nuclei derived from B-lymphocytes or sperm of *Homo sapiens *(HSA). 30 interphase nuclei were analyzed, each.

## Conclusion

The combination of MCB and S-FISH for a three-dimensional analysis of chromosome position in interphase nucleus is a powerful tool. The topological organization in interphase nucleus of hominoide has non-random way primarily driven by the gene density: #18, #19, #21 show a radial 3D-positioning, while #22 approximately equally localized in the peripheral and central territories of nucleus. Positioning of #18 homologues to each other has a random way in all studied species. The same holds true for homologues of #21 in HSA and HLA, but not in GGO. In the latter the orientation of #21 homologue shows the same non-random pattern like homologues of #22, i.e. they tend to be co-localized, presumably via the nucleolus. This suggestion is supported by the finding that in sperm, which do not have a nucleolus, only #22 has a different localization than in B-lymphocytes.

Further combined application of multicolor banding with three-dimensional analysis and immunohistochemistry will provide to a better understanding of interphase architecture in human and other primates.

## Methods

### Cell lines

Lymphoblastoid cell lines from human (*Homo sapiens *– HSA), gorilla (*Gorilla gorilla gorilla *– GGO) [17] and white-handed gibbon (*Hylobates lar *– HLA) were cultivated and cytogenetically prepared as previously reported [17,18]. All cell lines were karyotypically normal; GGO and HSA were female, HLA was a male.

### Human sperm

Human sperm sample was collected in a sterile container after 3 days of sexual abstinence from a fertile, 28 year-old man with normal seminal parameters and a normal karyotype. After liquefaction at room temperature, the sample was washed three times in 1 × phosphate-buffered saline (PBS) by centrifugation (5 min at 2000 rpm) and fixed in fresh fixative (1:3 glacial acetic acid: methanol) [19].

### Suspension-fluorescence in situ hybridization (S-FISH)

S-FISH on interphase cells prepared according to standard procedures [18] was done as previously reported [15] with some modifications. In short, the entire FISH procedure is performed on cell suspension and the interphase nuclei are placed on a polished concave slide as the final step of the procedure, just before the evaluation. It was shown before that by S-FISH it is possible to do 3D analyses on totally spherical interphase nuclei [15].

The main steps of S-FISH technique included: pepsin treatment (i.e. 475 μl H_2_O, 25 μl 0.2 N HCl, 0.005% pepsin), denaturation of DNA in interphase cells at 95°C, application of prepared DNA-probe containing 20 μg of COT1-DNA, hybridization over night at 37°C, washing in 0.4 SSC and 4 × SSC/0,2% Tween, blocking, detection and counterstaining in 0.5% DAPI-Vectashield (Vectashield; Vector, Burlingame, CA) [16]. As probes multicolor banding (MCB) sets for HSA #18, HSA #19, HSA #21 and HSA #22 were applied [14]. Images were captured on a Zeiss Axioplan microscope. Thirty interphase nuclei per chromosome were evaluated using software Cell-P (Olympus), rendering three-dimensional images and iso-surfaces that can be rotated freely and animated as well (Figs. 1, 2).

### Evaluation

The aforementioned three-dimensional images and iso-surfaces were evaluated concerning 3-dimensional measurements, such as position and distance of homologous chromosomes in Cell-P software. The interphase nucleus was zoned into two spheres, i.e. periphery (P) and center (C); 50% of the nucleus radius was defined as 'center'. Thus, either both homologues were located in the center (CC), in the periphery (PP) or both, in periphery and center (PC). Chromosomes located on the borderline between the compartments were classified as C or P in relation to where the majority of the chromsome body size was located. The localization of the homologue chromosomes to each other was estimated as 'close together' (t), 'near by each other' (n) or 'on the opposite sides of the nucleus' (o) – see Figure 1.

### Statistics

Statistical analysis was performed using Student's t – test, One Way ANOVA (Analysis of Variance) and χ^2 ^– test to determine significant differences of chromosome's arrangement in nucleus. Statistical significance was defined as p < 0.05.

## Competing interests

The authors declare that they have no competing interests.

## Authors' contributions

MM performed the 3-D FISH studies in the three hominoid species. MM, FH and SB did 3-D-FISH in human sperm. FP and SB provided and prepared the human sperm pellet. FH, KM and AW adapted the S-FISH protocol for MCB-probes. FP, AW, TL have been involved in drafting the manuscript and revising it critically for important intellectual content.
